# Task-Specific Transformer-Based Language Models in Health Care: Scoping Review

**DOI:** 10.2196/49724

**Published:** 2024-11-18

**Authors:** Ha Na Cho, Tae Joon Jun, Young-Hak Kim, Heejun Kang, Imjin Ahn, Hansle Gwon, Yunha Kim, Jiahn Seo, Heejung Choi, Minkyoung Kim, Jiye Han, Gaeun Kee, Seohyun Park, Soyoung Ko

**Affiliations:** 1 Department of Information Medicine Asan Medical Center Seoul Republic of Korea; 2 Big Data Research Center Asan Institute for Life Sciences Asan Medical Center Seoul Republic of Korea; 3 Division of Cardiology, Department of Information Medicine Asan Medical Center University of Ulsan College of Medicine Seoul Republic of Korea; 4 Division of Cardiology Asan Medical Center Seoul Republic of Korea; 5 Department of Medical Science Asan Medical Institute of Convergence Science and Technology, Asan Medical Center University of Ulsan College of Medicine Seoul Republic of Korea

**Keywords:** transformer-based language models, medicine, health care, medical language model

## Abstract

**Background:**

Transformer-based language models have shown great potential to revolutionize health care by advancing clinical decision support, patient interaction, and disease prediction. However, despite their rapid development, the implementation of transformer-based language models in health care settings remains limited. This is partly due to the lack of a comprehensive review, which hinders a systematic understanding of their applications and limitations. Without clear guidelines and consolidated information, both researchers and physicians face difficulties in using these models effectively, resulting in inefficient research efforts and slow integration into clinical workflows.

**Objective:**

This scoping review addresses this gap by examining studies on medical transformer-based language models and categorizing them into 6 tasks: dialogue generation, question answering, summarization, text classification, sentiment analysis, and named entity recognition.

**Methods:**

We conducted a scoping review following the Cochrane scoping review protocol. A comprehensive literature search was performed across databases, including Google Scholar and PubMed, covering publications from January 2017 to September 2024. Studies involving transformer-derived models in medical tasks were included. Data were categorized into 6 key tasks.

**Results:**

Our key findings revealed both advancements and critical challenges in applying transformer-based models to health care tasks. For example, models like MedPIR involving dialogue generation show promise but face privacy and ethical concerns, while question-answering models like BioBERT improve accuracy but struggle with the complexity of medical terminology. The BioBERTSum summarization model aids clinicians by condensing medical texts but needs better handling of long sequences.

**Conclusions:**

This review attempted to provide a consolidated understanding of the role of transformer-based language models in health care and to guide future research directions. By addressing current challenges and exploring the potential for real-world applications, we envision significant improvements in health care informatics. Addressing the identified challenges and implementing proposed solutions can enable transformer-based language models to significantly improve health care delivery and patient outcomes. Our review provides valuable insights for future research and practical applications, setting the stage for transformative advancements in medical informatics.

## Introduction

### Background

Transformer models have revolutionized natural language processing (NLP) with their exceptional state-of-the-art performance in various applications such as conversation, translation, text classification, and text generation. A transformer model is a type of deep learning model designed to process and generate sequences of data, such as text. The key innovation of transformer models is the self-attention mechanism, which allows the model to weigh the importance of different words in the input sequence, regardless of their position. Self-attention allows the model to focus on different parts of an input sequence simultaneously, rather than processing the sequence in a fixed order. This mechanism enables the model to capture complex patterns and relationships within the context more effectively than previous models, which is particularly useful for understanding and generating natural language. These models hold significant promise for the health care sector, addressing clinical challenges and unlocking new opportunities in medical informatics (eg, disease prediction, clinical decision support, and patient interaction).

Since the introduction of the transformer model by Google [1] in 2017, it has become the foundation for various pretrained language models (PLMs). PLMs are transformer models that have been initially trained on a large text corpus before being fine-tuned for specific tasks. This pretraining allows the models to leverage vast amounts of unstructured data to improve their performance in various NLP tasks. Two of the most widely used PLM architectures in medical research are Generative Pre-trained Transformer (GPT) and Bidirectional Encoder Representations from Transformers (BERT). GPT is designed to generate coherent text based on a given input, making it useful for tasks like dialogue generation [2]. BERT, on the other hand, is designed to understand the context of words in a sentence from both directions, making it highly effective for tasks like question answering and text classification [3]. Transformer-based language models have revolutionized the field of NLP and continued to advance the state-of-the-art in NLP with their impressive performance.

Despite the success of transformer-based language models in many domains, there is a significant gap in comprehensive reviews specifically focused on their application in the health care domain. In health care, transformer-based language models have been used for crucial tasks such as disease prediction, decision-making, and image analysis [4]. With the abundance of free text sources, such as medical documentation in free text, including social media, electronic medical records (EMRs), physician-patient conversations, and online encyclopedias, more significant challenges to language models are needed. The application of NLP in health care is not without controversy, particularly concerning data privacy, ethical implications, and the integration of artificial intelligence (AI) systems into clinical practices. Debates continue about the extent to which AI can replace human judgment, the transparency of AI decision-making processes, and the potential biases in AI models trained on unbalanced datasets. By addressing these concerns, our paper contributes to the timely and critical discourse on the responsible deployment of transformer-based language models in health care, emphasizing the need for transparency, fairness, and ethical considerations in AI development.

### Objective

The objective of this paper is to provide a comprehensive scoping review of task-specific transformer-based language models in health care. By focusing on models pretrained on medical corpora, we aim to address the gap in existing literature where detailed surveys specifically tailored to health care applications are lacking. We seek to highlight the strengths, limitations, and potential of these models, offering valuable insights for future research and practical applications in medical informatics.

### Related Work

While many review studies of NLP have been conducted in the medical field [5-13], on transformer-based language models [14-20], and in health-related domains [21-25], comprehensive surveys and broader and up-to-date transformer-based language models in health care are lacking, leaving a gap in understanding their full potential and limitations. Pandey et al [5] introduced RedBERT, a model focusing on topic discovery and deep sentiment classification of COVID-19 online discussions, demonstrating the application of NLP in understanding public health concerns. Iroju and Olaleke [6] conducted a systematic review of NLP applications, identifying key areas where NLP can enhance clinical decision-making and patient care. Similarly, Locke et al [7] provided a comprehensive overview of NLP in medicine, emphasizing the potential of NLP technologies in transforming medical practice. Adyashreem et al [8] surveyed various NLP techniques in the biomedical field, shedding light on how these techniques can be applied to biomedical text for improved information extraction and analysis. Wang et al [9] reviewed the application of NLP in clinical medicine, highlighting the advancements and challenges in integrating NLP with clinical workflows.

Khanbhai et al [11] applied NLP and machine learning techniques to patient experience feedback, providing insights into patient satisfaction and areas for improvement in health care services. Casey et al [12] focused on NLP applications in radiology reports, identifying how NLP can streamline the interpretation and reporting of radiological findings. Zhou et al [13] discussed the broader applications of NLP for smart health care, envisioning a future where NLP-driven systems enhance patient care and operational efficiency.

In the realm of transformer-based language models, Zhang et al [14] surveyed their applications in bioinformatics, highlighting how these models have advanced the analysis of biological data. Yang [15] and Lin et al [16] explored the progress and applications of transformer models in Korean and general NLP tasks, respectively, highlighting their growing importance and versatility. Chitty-Venkata et al [17] reviewed neural architecture search for transformers, underscoring the potential of these models in optimizing NLP tasks. Gillioz et al [18] provided an overview of transformer-based models for various NLP tasks, illustrating their adaptability and efficiency. Han et al [19] focused on multimodal pretrained models, emphasizing their capability to handle diverse data types, including text, image, and audio. Greco et al [20] and Albalawi et al [21] discussed transformer models’ applications in mental health and Arabic social media, respectively, highlighting their potential in understanding and addressing specific health-related issues. Kalyan et al [22] and Shamshad et al [23] provided comprehensive surveys on biomedical PLMs and their applications in medical imaging, respectively, showcasing the transformative impact of transformers in these fields.

Our review categorizes these models into 6 key tasks: dialogue generation, question answering, summarization, text classification, sentiment analysis, and named entity recognition (NER). Ultimately, advancements in transformer-based language models hold the promise of significantly transforming health care delivery and improving patient outcomes. By enabling more accurate disease prediction, enhancing clinical decision support, and facilitating better patient-provider communication, these models can lead to more efficient, effective, and personalized health care. Our review underscores the broader implications of these technologies, advocating for continued research and development to harness their full potential in revolutionizing medical informatics and patient care.

## Methods

### Information Source and Search Strategy

We followed the Cochrane scoping review protocol to conduct and map the available literature in an efficient and systematic approach. This method involves defining the research question, identifying relevant studies, selecting studies based on predefined criteria, charting data, and summarizing the results to clarify key concepts and identify research gaps [24].

Our research team (mainly HNC and TJJ) conducted a comprehensive literature review for identifying studies in the field that met the inclusion and exclusion criteria. The screening and selection of papers were conducted by 2 independent reviewers (HNC and TJJ). Initially, titles and abstracts were screened to identify relevant studies. Full texts of potentially eligible studies were then reviewed to ensure they met the inclusion criteria. Disagreements between reviewers were resolved through discussion and consensus, with a third reviewer (YHK) consulted if necessary. Our literature search was conducted across several scientific databases, including Google Scholar and PubMed, which were selected for their comprehensive coverage of relevant journals and peer-reviewed studies in the medical and academic fields. We covered publications from January 01, 2017, to September 30, 2024, and used specific combinations of keywords and Boolean operators, such as “transformer-based AND language models AND medical domain,” “health care AND language models,” “NLP AND medicine AND survey,” and “GPT AND BERT AND health care.” Data extraction involved summarizing key findings, model names, and training datasets. The extracted data were cross-verified by both reviewers to ensure accuracy and consistency. Any discrepancies were resolved through discussion.

**Figure 1:**
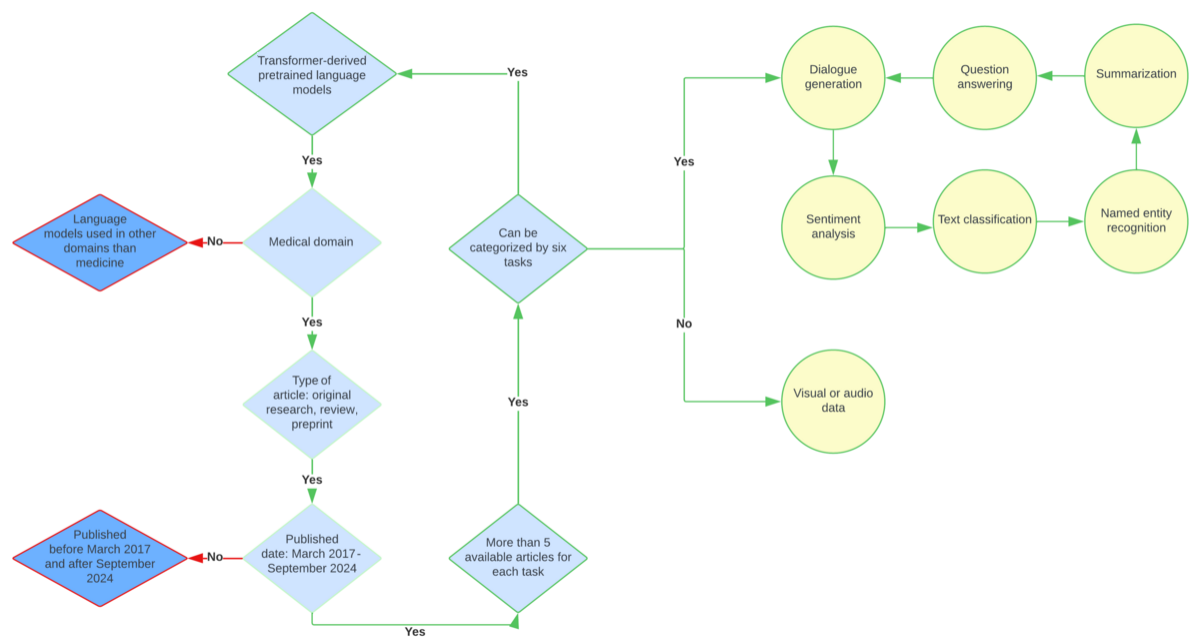
Article filtering process with inclusion criteria.

We included studies that involved transformer-derived models applied to medical tasks, were published in peer-reviewed journals, and were written in English. The exclusion criteria involved studies focusing solely on non-text data (eg, audio, image, and video) or those not meeting the inclusion requirements. The selection of tasks (dialogue generation, question answering, summarization, text classification, sentiment analysis, and NER) was based on their critical role in advancing health care applications of transformer models. The specific process is illustrated in Figure 1, with details of each stage of filtering from the initial identification of articles to the final selection. The inclusion criteria were rigorously applied at each step, beginning with the screening of titles and abstracts, followed by a full-text review, and culminating in the inclusion of studies that met all predefined criteria. This methodical approach allowed us to compile a comprehensive and focused set of articles for our scoping review, ensuring that our findings are both robust and reliable.

These tasks cover a wide range of functionalities essential for improving clinical workflows, enhancing patient interactions, and facilitating efficient information retrieval and analysis, making them vital for the advancement of transformer-based language models in the medical domain. Languages and model types were chosen to represent a diverse range of medical contexts and applications.

**Figure 2:**
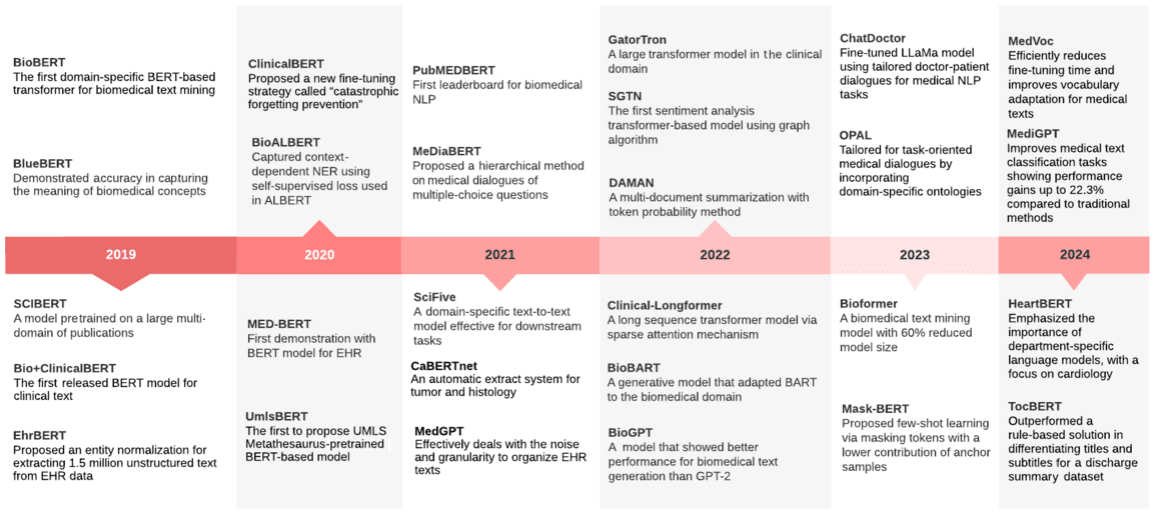
Timeline of significant transformer-based models in health care. EHR: electronic health record; NER: named entity recognition; NLP: natural language processing.

In this section, studies that have used language models in health care applications were examined. Based on the literature review, Table 1 provides a comprehensive list of transformer-based models applied in the medical domain, comparing each task based on the authors, model name, training dataset, PLM model, key metric, score, and purpose or findings of the study. These English-written PLMs in the health care domain were categorized into 6 distinct tasks, namely dialogue generation, question answering, summarization, text classification, sentiment analysis, and NER. The articles within each task are listed in no sequential order. In Figure 2, the evolution timeline of transformer-based language models provides an overview of significant models that have been developed for use in medicine. It illustrates key milestones and the deployment criteria used to guide the inclusion of studies in our review. This historical context provides a foundation for understanding the methodological choices made in our scoping review. This visual representation highlights the emergence of models over time and their increasing significance in health care applications. We provide insights into the progress made in this field and anticipate future advancements by tracking the development of these models.

**Table 1:** Summary of the applications of pretrained language models subdivided into tasks.

Task and author (year)			Model name		Training dataset		PLM^a^ model		Key metric			Score (%)		Key findings
**Conversation**														
	Varshney et al [26], 2023	Medical Entity Prediction (MEP)		UMLS		BERT		Accuracy			85		Integrated triples from knowledge graphs to enhance medical predictions using a large pretrained model.	
	Yuan et al [27], 2022	BioBART		PubMed		BART		Rouge-2			65		Adapted and improved biomedical context understanding through advanced generative techniques.	
	Zhao et al [28], 2022	MedPIR		MedDG, MedDialog		BERT, GPT		F1			82		Used a knowledge-aware dialogue graph encoder (KDGE) and recall-enhanced generator (REG) to improve clinical responses.	
	Chen et al [29], 2023	OPAL		Wikipedia, WOZ, CamRest676		BART		BLEU			21.5		Tailored for task-oriented medical dialogues by incorporating domain-specific ontologies.	
	Liang et al [30], 2021	MKA-BERT-GPT		MedDG, MedDialog-CN		BERT, GPT		Relevance improvement			15		First scalable model to integrate a medical knowledge graph into a large pretrained model, enhancing biomedical understanding.	
	Compton et al [31], 2021	MEDCOD		KB, doctor edits		GPT-3		Emotive accuracy			90		Generated diverse, emotive, and empathetic sentences for health care interactions.	
	Li et al [32], 2023	ChatDoctor		5000 doctor-patient conversations		LlaMA		Precision, recall, F1			83.7, 84.5, 84.1		Fine-tuned LLaMa model using tailored doctor-patient dialogues for medical NLP^b^ tasks.	
	Tang et al [33], 2023	-w terms+AL		MedDialog		BART		Annotation accuracy			87		Automated large-scale medical conversation text annotation with terminology extraction.	
	Zeng et al [34], 2020	Transformer-DST		MultiWOZ		BERT		DST accuracy			54.6		Proposed a transformer-based framework using a flat encoder-decoder architecture for dialogue state tracking in medical contexts.	
	Suri et al [35], 2021	MeDiaBERT		MeDiaQA		BERT		Accuracy			64.3		Employed a hierarchical approach to medical dialogue analysis, including multiple-choice question answering.	
	Phan et al [36], 2021	SciFive		PubMed		T5		Accuracy			86.6		A medical T5 text-to-text model effective for various clinical downstream tasks.	
	Wu et al [37], 2023	PMC-LLaMA		PubMed, 30K Medical Books		LlaMA		Accuracy			64.43		Transitioned a general-purpose model to a high-performing medical language model via comprehensive fine-tuning, achieving state-of-the-art performance in medical question answering.	
	Zhang et al [38], 2023	HuatuoGPT		Huatuo26M		GPT		BLEU, ROUGE, distinct			25.6, 27.76, 93		Chinese health care LLM: Tailored for the Chinese medical domain, providing state-of-the-art results in medical consultation tasks.	
**Question answering**														
	Lee et al [39], 2019	BioBERT		PubMed, EHR^c^, clinical notes, patents		BERT		MRR improvement			12.24		First domain-specific BERT-based model for biomedical text mining, outperforming standard BERT in medical tasks.	
	Luo et al [40], 2023	BioGPT		PubMed		GPT		Accuracy			78.2		Pretrained on a 15M PubMed corpus, this model outperforms GPT-2 in biomedical text generation.	
	Shin et al [41], 2020	BioMegatron		Wikipedia, news, OpenWebtext		Megatron-LM		Bias			40		Enhanced the representation of biomedical entities across a large corpus for better entity understanding.	
	Rasmy et al [42], 2020	MED-BERT		Cerner Health Facts, Truven		BERT		AUC^d^			20 boosts		First proof-of-concept BERT model for integrating electronic health records.	
	Yasunaga et al [43], 2022	LinkBERT		Wikipedia		BERT		Improvement			5		Effective in multi-hot reasoning and few-shot question answering by linking documents.	
	Michalopoulos et al [44], 2020	UmlsBERT		MIMIC-III		BERT		F1			86		Learned the association of clinical terms within the UMLS metathesaurus.	
	Zhang et al [45], 2021	SMedBERT		ChineseBLUE		BERT		Accuracy			78		Introduced a mention-neighbor hybrid attention model for heterogeneous medical entity information.	
	Yang et al [46], 2022	ExKidneyBERT		EMR^e^		BERT		Accuracy			95.8		A specialized model focused on renal transplant-pathology integration.	
	Mitchell et al [47], 2021	CaBERTnet		Pathology reports		BERT		Accuracy			85		An automatic system for extracting tumor sites and histology information.	
	Trieu et al [48], 2021	BioVAE		PubMed		SciBERT, GPT2		VAE			72.9		First large-scale pretrained language model using the OPTIMUS framework in the biomedical domain.	
	Khare et al [49], 2021	MMBERT		COntext (ROCO)		BERT		Accuracy			72		Proposed masked language modeling for radiology text representations.	
	Yasunaga et al [43], 2022	BioLinkBERT		Wikipedia, Book Corpus		BERT		BLURB			84		A novel linking method for predicting document relations in pretraining models.	
	Nguyen et al [50], 2022	SPBERTQA		ViHealthQA		SBERT		Mean average precision			69.5		A 2-step question answering system addressing linguistic disparities with BM25 and Sentence BERT.	
	Luo et al [51], 2023	BioMEDGPT		PubMed		GPT				Accuracy	76.1		First multimodal GPT capable of aligning biological modalities with human language for medical text analysis.	
	Toma et al [52], 2023	Clinical Camel		PubMed, USMLE, MedMCQA		LlaMA-2		Five-shot accuracy			74.3, 54.3, 47.0		A model that outperforms GPT-3.5 by using efficient fine-tuning techniques.	
	Han et al [53], 2023	MedAlpaca		Medical flash cards, Wikidoc		Alpaca		Accuracy			21.1-24.1		Highlighted privacy protection in medical artificial intelligence and demonstrated significant performance enhancements in medical certification exams through fine-tuning.	
	Singhal et al [54], 2023	MedPaLM-2		MedMCQA, MedQA, PubMedQA, MMLU		PaLM		Accuracy			67.6		Instruction prompt tuning undergoes rigorous human evaluation to assess harm avoidance, comprehension, and factual accuracy.	
	Chen et al [55], 2023	MEDITRON		PubMED		LlaMA-2		Accuracy			79.8		Achieved 6% improvement over the best public baseline and 3% gain over fine-tuned Llama-2 models.	
**Summarization**														
	Yan et al [56], 2022	RadBERT		Open-I chest radiograph report		BERT		Accuracy, F1			97.5, 95		Adapted a bidirectional encoder representation for radiology text.	
	Du et al [57], 2020	BioBERTSum		PubMed		BERT		ROUGE-L			68		Introduced the first transformer-based model for extractive summarization in the biomedical domain.	
	Li et al [58], 2022	Clinical-Longformer & Clinical-Big Bird		MIMIC-III		Longformer, Big Bird		F1			97		Reduced memory usage through sparse attention in a long-sequence transformer.	
	Moro et al [59], 2022	DAMEN		MS2		BERT, BART		Accuracy			75		Developed a multi-document summarization method using token probability.	
	Chen et al [60], 2020	AlphaBERT		HER (NTUH-iMD)		BERT		Accuracy			69.3		Designed a diagnoses summarization model based on character-level tokens.	
	Alsentzer et al [61], 2019	Bio+Clinical BERT		MIMIC-III		BERT		F1			11 improvements		Released the first BERT-based model specifically for clinical text.	
	Cai et al [62], 2021	ChestXRayBERT		MIMIC		BERT		Accuracy			73		Automatically generates abstractive summarization of radiology reports.	
	Yalunin et al [63], 2022	LF2BERT		UMLS, EHR		BERT		ROUGE-1 F1, ROUGE-2 F1, ROUGE-L F1			67, 56.4, 64.5		Developed a neural abstractive model for summarizing long medical texts.	
	Balde et al [64], 2024	MEDVOC		PubMed, BioASQ, EBM		GPT		ROUGE			51.49, 47.54, 19.51		Efficiently reduced fine-tuning time and improved vocabulary adaptation for medical texts.	
**Text classification**														
	Yang et al [65], 2022	GatorTron		Clinical notes, UF Health clinical corpus MIMIC-III, PubMed, Wikipedia		GPT		Pearson correlation			89		Outperformed previous biomedical and clinical domain models.	
	Gu et al [66], 2021	PubMEDBERT		PubMed		BERT		BLURB			81.2		Established a leaderboard for biomedical NLP, with robustness against noisy and incomplete biomedical text.	
	Huang et al [67], 2020	ClinicalBERT		EHR (clinical notes)		BERT		Accuracy, precision, recall, AUROC^f^, AUPRC^g^			72.7, 37.6, 54.2, 74.2, 42.0		Introduced “catastrophic forgetting prevention” and generated visualized interpretable embeddings.	
	Gupta et al [68], 2022	MatSciBERT		Wikipedia, clinical database, Book Corpus		BERT		F1			81.5		Effective transformer model for scientific text analysis.	
	Fang et al [69], 2023	Bioformer		PubMed, PMC		BERT		Performance, speed			60 reduced model size, 2-3× speed		Reduced model size by 60% for biomedical text mining.	
	Gururangan et al [70], 2020	BioMed-RoBERTa		CHEMPROT, PubMed		RoBERTa		F1			83.4		Proposed domain and task-adaptive pretraining with a data selection strategy.	
	Liao et al [71], 2023	Mask-BERT		PubMed, NICTA-PIBOSO, symptoms		BERT		Accuracy, F1, PR-AUC^h^			91.8, 89.6, 93.1		Improved a BERT-based model for multiple tasks with masked input text.	
	He et al [72], 2022	KG-MTT-BERT		EHR		BERT		Accuracy			82		Developed a model for multi-type medical tests using a knowledge graph.	
	Yang et al [73], 2023	TransformEHR		EHR		BERT		AUROC, AUPRC			81.95, 78.64		Set a new standard in clinical disease prediction using longitudinal EHRs.	
	Pedersen et al [74], 2023	MeDa-BERT		EMR		BERT		Accuracy			86.7-97.1		Tailored embeddings for Danish medical text processing.	
	Hong et al [75], 2023	SCHOLARBERT		Public resource		BERT		F1			85.49		Leveraged a public resource-driven dataset for scientific NLP.	
	Abu Tareq Rony et al [76], 2024	MediGPT		Illness dataset		GPT		Accuracy, F1			90.0, 88.7		Improved medical text classification tasks showing performance gains up to 22.3% compared to traditional methods.	
**Sentiment analysis**														
	Ji et al [77], 2021	MentalBERT/MentalRoBERTa		Reddit		BERT, RoBERTa		F1, recall			81.76, 81.82		A pretrained masked model designed for mental health detection.	
	Taghizadeh et al [78], 2021	SINA-BERT		Self-gathered collection of texts from online sources		BERT		Precision, recall, macro F1, accuracy			94.91, 94.63, 94.77, 96.14		Developed a pretrained language model for the Persian medical domain.	
	AlBadani et al [79], 2022	SGTN		SemEval, SST2, IMDB, Yelp		BERT		Accuracy			80		Proposed the first sentiment analysis model using a transformer-based graph algorithm.	
	Pandey et al [80], 2021	RedBERT		Reddit		BERT		Accuracy			86.05		Introduced a sentiment classification method from web-scraped data.	
	Palani et al [81], 2021	T-BERT		Twitter		BERT		Accuracy			90.81		Designed a sentiment classification method for microblogging platforms.	
	Mao et al [82], 2022	AKI-BERT		MIMIC-III		BERT		AUC, precision, recall/sensitivity, F1, specificity, negative predictive value			74.7, 35.6, 61.9, 45.2, 76.8, 90.7		Created a BERT model for predicting acute kidney injury.	
	Chaudhary et al [83], 2020	TopicBERT		Ohsumed		BERT		Cost optimization			70		Improved computational efficiency by combining topic and language models for fine-tuning.	
	Qudar et al [84], 2020	TweetBERT		NCBI, BC5CDR, BIOSSES, MedNLI, Chemprot, GAD, JNLPBA		BERT		F1			87.1		Achieved state-of-the-art performance on biomedical datasets using Twitter data for pretraining.	
	Wouts et al [85], 2021	BelabBERT		DBRD		RoBERT		Accuracy			95.9		Developed a Dutch language model for psychiatric disease classification.	
**Named entity recognition**														
	Li et al [86], 2020	BEHRT		EHR		BERT		Accuracy			81		Interpretable model for multi-heterogeneous medical concepts.	
	Shang et al [87], 2019	G-BERT		EHR		BERT		Jaccard, PR-AUC, F1			45.7, 69.6, 61.5		The first pretraining method for medication recommendation in the medical domain.	
	Lentzen et al [88], 2022	BioGottBERT		Wikipedia, drug leaflets from AMIce, LIVIVO		RoBERTa, GottBERT		Accuracy			78		Introduced the first transformer model for German medical texts.	
	Davari et al [89], 2020	TIMBERT		PubMed		BERT		Precision, recall, F1			90.5, 91.2, 90.9		Developed a BERT-based model for automated toponym identification.	
	Peng et al [90], 2019	BlueBERT		PubMed, MIMIC-III		BERT		Masked token score			77.3		Demonstrated strong generalization ability across biomedical texts and cross-lingual tasks.	
	Miolo et al [91], 2021	ELECTRAMed		NCBI		BERT		Precision, recall, F1			85.9, 89.3, 87.5		The first ELECTRA-based model for the biomedical domain.	
	Khan et al [92], 2020	MT-BioNER		BC2GM, BC5CDR, NCBI-Disease		BERT		Precision, recall, F1			88.4, 90.52, 89.5		A multi-task transformer model for slot tagging in the biomedical domain.	
	Naseem et al [93], 2020	BioALBERT		PubMed, PMC		BERT		Precision, recall, F1			97.4, 94.4, 95.9		Trained on large biomedical corpora using ALBERT for biomedical text mining.	
	Yang et al [94], 2021	BIBC		Textbooks, research papers, clinical guidelines		BERT		Accuracy			78		Designed a new architecture for processing long text inputs in diabetes literature.	
	Martin et al [95], 2020	CamemBERT		Wikipedia		RoBERTa		Accuracy			85.7		Developed the first monolingual RoBERTa model for French medical text.	
	Kraljevic et al [96], 2021	MedGPT		EHR		GPT		Precision			64		Efficiently handled noise in EHR data using NER and MedCAT.	
	Li et al [97], 2019	EhrBERT		EHR		BERT		F1			93.8		Proposed an entity normalization technique for 1.5 million EHR notes.	
	Gwon et al [98], 2024	HeartBERT		EMR		BERT		Accuracy			74		Emphasized the importance of department-specific language models, with a focus on cardiology.	
	Mannion et al [99], 2023	UMLS-KGI-BERT		UMLS		BERT		Precision			85.05		Introduced a graph-based learning method with masked-language pretraining for clinical text extraction.	
	Schneider et al [100], 2023	CardioBERTpt		EHR		BERT		FL-score			83		Specialized in extracting Portuguese cardiology terms, demonstrating that data volume and representation improve NER performance.	
	Saleh et al [101], 2024	TocBERT		MIMIC-III		BERT		F1			84.6		Outperformed a rule-based solution in differentiating titles and subtitles for a discharge summary dataset.	

^a^PLM: pretrained language model.

^b^NLP: natural language processing.

^c^EHR: electronic health record.

^d^AUC: area under the curve.

^e^EMR: electronic medical record.

^f^AUROC: area under the receiver operating characteristic curve.

^g^AUPRC: area under the precision-recall curve.

^h^PR-AUC: precision-area under curve.

## Results

### Selected Studies

**Figure 3:**
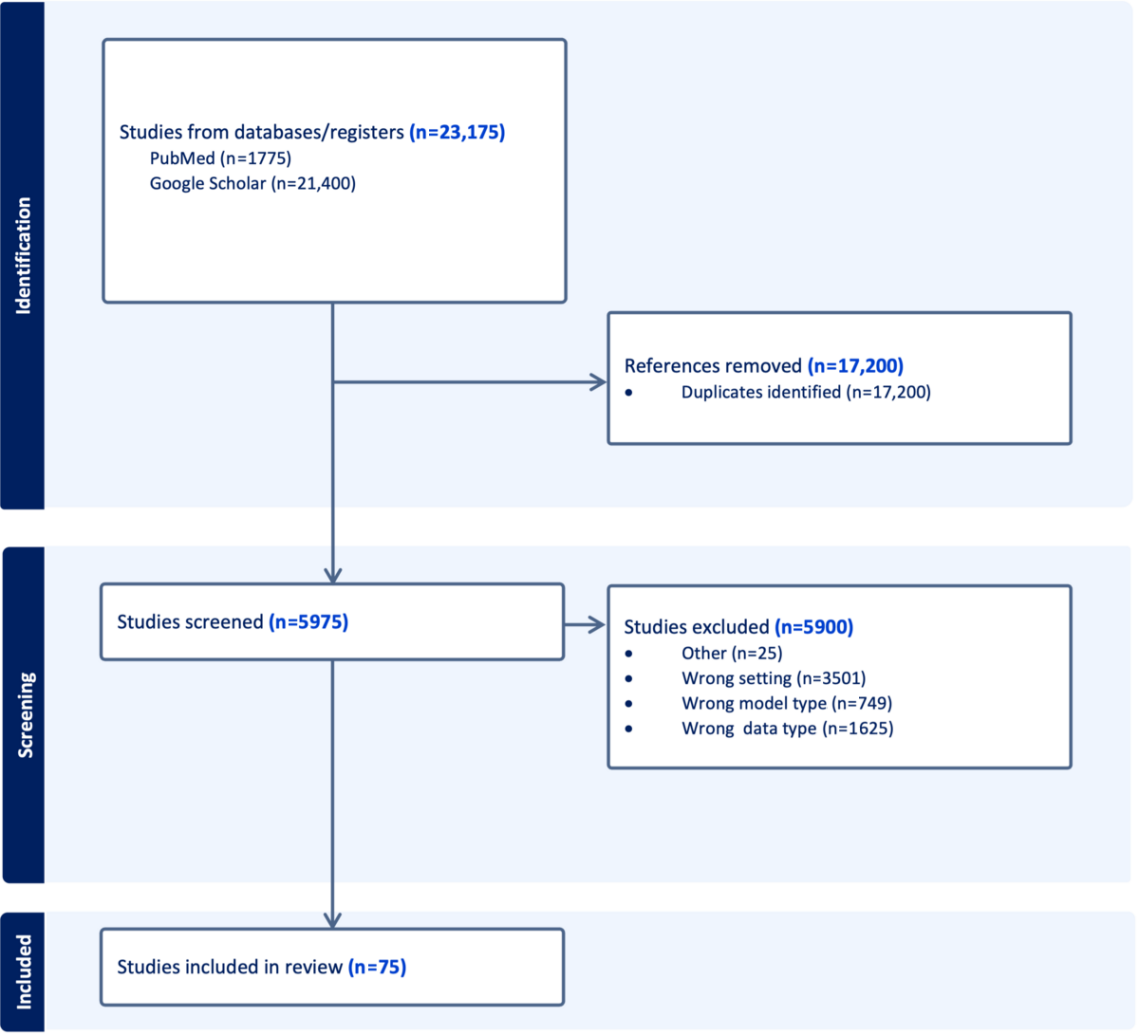
PRISMA (Preferred Reporting Items for Systematic Reviews and Meta-Analyses) flow diagram for the review process.

A total of 75 models were identified through our comprehensive review. The PRISMA (Preferred Reporting Items for Systematic Reviews and Meta-Analyses) flowchart is presented in Figure 3. The PRISMA checklist is presented in Multimedia Appendix 1. These papers encompass various research areas related to transformer-based models and their applications in the medical domain. The selection of these papers was based on predefined inclusion criteria, ensuring the relevance of each study to the scope of our review.

### Applications of Language Models in Health Care: Task-Specific

#### Dialogue Generation

Conversation generation generates responses to a given dialogue. GPT models, including DialoGPT and DialogBERT, can effectively generate human-like dialogues based on large corpora and contextualized representations of text [25,102-105]. In the medical domain, conversation generation focuses on developing conversations related to medical information [106]. Chatbots in health care can be classified into 6 types: screening and diagnosis, treatment, monitoring, support, workflow efficiency, and health promotion. These tasks involve aiding patient consultation, acting as a physician’s decision support system, collaborating with interdisciplinary research, and providing care instructions and medical education [107,108].

The key models are MEP, BioBART, MedPIR, MEDCOD, Transformer-DST, MeDiaBERT, ChatDoctor, and SciFive.

Research efforts on conversation generation in medicine have also incorporated knowledge graphs. MKA-BERT-GPT was the first scalable work to integrate a medical knowledge graph mechanism into a large pretrained model. Meanwhile, MedPIR proposed a recall-enhanced generator framework by using a knowledge-aware dialogue graph encoder to strengthen the relationship between the user input and the response via past conversation information [28,30]. They achieved an F1 score of 82% and a bilingual evaluation understudy (BLEU) score of 21.5. Varshney et al [26] proposed the Masked Entity Dialogue (MED) model to train smaller corpora texts, addressing a 10% improvement in entity prediction accuracy for the problem of local embeddings in entity embeddings by incorporating conversation history into triples in the graphs, resulting in an automatic prediction of the medical entities model.

On the other hand, MEDCOD [31] used the GPT pretrained model to integrate emotive and empathetic aspects into the output sentences, which further imitates a human physician–like feature to better communicate with patients. The Transformer-DST [34] model addresses dialogue state tracking, optimizing state operation prediction, and value generation with high accuracy by suggesting to ask the DST model to consider the whole dialogue and the previous state. Moreover, MeDiaBERT [35], using a hierarchical approach, achieves 63.8% accuracy in multiple choice medical queries by building a transformer encoder contained within another in a hierarchical manner.

BioBART [27], a BART-based model, used patient descriptions and conversation histories as input for the model to autoregressively generate replies to user inputs. The model outperformed the BART model by 1.71 on Rouge-2 with a BLEU score of 4.45, and pretraining on PubMed abstracts supported the model’s performance. OPAL [29] and -w terms+AL models also used BART for pretraining. OPAL’s proposed method involves 2 phases: pretraining on large-scale contextual texts with structured information extracted using an information-extracting tool and fine-tuning the pretrained model on task-oriented dialogues. The results showed a significant performance boost, overcoming the problems created by annotated data with large structured dialogue data. Recently, the -w terms+AL model proposed a framework for improving dialogue generation by incorporating domain-specific terminology through an automatic terminology annotation framework using a self-attention mechanism [33].

While other models are based on BERT, GPT, or BART, ChatDoctor [32], SciFive [36], and PMC-LLaMA [37] use LLaMA or T5 PLMs. To improve accuracy and provide informed advice in medical consultations, ChatDoctor used Meta’s open-source LLaMA [109], which was fine-tuned using real-world patient-physician conversations and autonomous knowledge retrieval capabilities, achieving 91.25% accuracy. SciFive, a Text-To-Text Transfer Transformer–based model, was pretrained on large biomedical corpora, indicating its significant potential for learning large and extended outputs. The SciFive model was trained using a maximum likelihood objective with “teacher forcing” [110] for multi-task learning by leveraging task-specific tokens in the input sequence. Both models outperformed previous baseline methods.

More recently, the HuatuoGPT model [38], specifically tailored for the Chinese medical domain, provided state-of-the-art results in medical consultation tasks.

#### Question Answering

The question-answering task involves answering questions posed by users based on the texts in documents. It aims to generate an accurate response that directly answers the question input, contributing to clinical decision-making, medical education, and patient communication. Allowing physicians and researchers to obtain valuable answers quickly from electronic health records (EHRs) and various medical literature will effectively reduce the time and effort required when the procedure is done manually. While the dialogue generation and question-answering tasks both involve providing answers, the former focuses on generating responses within a conversation, whereas the latter focuses on developing specific answers to user questions.

The key models are BioBERT, BioGPT, BioMegatron, Med-BERT, UmlsBERT, SMedBERT, and BioVAE.

BERT-based language models have become increasingly popular in biomedical text mining as they can understand the context and generate accurate predictions. BioBERT [39], the first domain-specific BERT-derived transformer language model for biomedical text mining applications, achieved 89% accuracy on the MedQA dataset and outperformed BERT in medical text applications. A BioMegatron model [41], based on Megatron-LM [111], was also experimented on a question-answering task, and it was found that the domain and task-specific language model affected the overall performance rather than the model size. Shin et al [41] found that model size is not closely related to the performance rate, but rather the domain and task-specific language model affects the overall performance. Med-BERT [42], another BERT-inspired model, improved the prediction accuracy by 20% in disease prediction studies by pretraining on EHR datasets.

More recently, researchers have built BERT-based models for specific domains and tasks [112]. UmlsBERT [44] first built a semantic embedding linking concepts with the words in the UMLS Metathesaurus and proposed multi-label loss function–masked modeling. SMedBERT [45] also presented a similar approach with the knowledge semantic representation but structured the neighboring entities to learn heterogeneous information. UmlsBERT and SMedBERT enhanced performance, with F1 scores of 84% and 86%, respectively. Similarly, LinkBERT and BioLinkBERT [43] incorporated ontological knowledge to better understand a linking system between entities in the corpus. LinkBERT used a multi-task learning framework on several related tasks simultaneously to extract relations between entities in the corpus more effectively. ExKidneyBERT, CaBERTnet, and MMBERT extracted more precise answers from individual departmental reports [46,47,49].

On the other hand, BioVAE [48] used the OPTIMUS framework pretrained with SciBERT [113] and GPT-2 [114,115] and outperformed the baseline models on biomedical text mining. To address the issues on linguistic disparity, SPBERTQA [50] proposed a 2-stage multilingual language model pretrained on the SBERT model [116] to reply to user questions using multiple negative ranking losses with Bert multilingual 25.

However, previous studies using the BERT structure are a better fit for understanding the context, rather than generating texts. To this end, BioMedLM, a GPT architecture model, was built mainly for biomedical question-answering tasks [117] in recent studies of question-answering benchmarks and achieved 50% accuracy on summarizations of the patient’s quest even in real situations with fewer data. BioGPT [40] applied a 2-step fine-tuning method to remove the noise in data and achieved 6.0% improved results compared with BioLinkBERT in the medical domain for question-answering tasks.

Recent studies have introduced significant advancements, such as BioMEDGPT [51], the first multimodal GPT for aligning biological data with human language, achieving 76.1% accuracy. Clinical Camel [52], using LLaMA-2, demonstrated superior performance with 5-shot accuracy ranging from 47.0% to 74.3%, outperforming GPT-3.5. MedAlpaca [53] focused on privacy and medical certifications, attaining 21.1%-24.1% accuracy. MedPaLM-2 [54] reached 67.6% accuracy through instruction prompt tuning, and MEDITRON [55] achieved 79.8% accuracy, marking a 6% improvement upon existing models and setting a new benchmark.

#### Summarization

For many years, the medical field has suffered from the challenge of finding efficient and rapid access to understanding the fast-growing and immensely increasing amount of data formation. The key to timely and efficient clinical workflow is providing automatic summarization in clinical text. Summarization in health care is an important technique in NLP as it automatically summarizes the medical contexts into a concise summary of text. Summarization can be applied to medical records, literature, clinical trial reports, and other types of medical texts that aim to provide clinical providers with quick access to relevant information, without the need to skim through lengthy documents. Overall summarization can aid clinicians with decision-making through effective and prompt communication during the physician-patient meeting, as well as knowledge discovery for medical research [57].

The key models are BioBERTSum, AlphaBERT, ClinicalBertSum, ChestXRayBERT, RadBERT, LF2BERT, and DAMEN.

To alleviate the problems of biomedical literature summarization, which can have difficulties in learning sentence and document-level features, Du et al [57] proposed the first PLM for medical extractive summarization application called BioBERTSum. BioBERTSum captures a domain-aware token and sentence-level context by using a sentence position embedding mechanism that inserts structural information into a vector representation. It achieved a ROUGE-L score of 0.68, outperforming standard BERT models. AlphaBERT [60] proposed a diagnostic summary extractive model using a character-level token to reduce the model size and achieved a ROUGE-L score of 0.693, reducing the burden of physicians in the emergency department regarding reading complex discharge notes of patients.

To better use clinical notes, ClinicalBertSum [118] used the ClinicalBERT, SciBERT, and BertSum models during the fine-tuning and summarization process to automatically extract summaries from clinical abstracts. Similarly, ChestXRayBERT used BERT to perform an automatic abstractive summarization on radiology reports [62], with ROUGE-1 scores of 0.70 and 0.73, respectively. RadBERT [56], which was fine-tuned for radiology report summarization, achieved 10% fewer annotated sentences during the training, demonstrating the benefit of domain-specific pretraining to increase the overall performance.

LF2BERT [63] applied a Longformer neural network and BERT in an encoder-decoder framework to process longer sequence inputs and performed better than human summarization, according to doctors’ evaluations. DAMEN [59] used BERT together with BART to discriminate important topic-related sentences in summarization, outperforming previous methods to summarize multiple medical literature via the token probability distribution method. The proposed probabilistic method selected only related significant chunks of information and then provided the probabilities of the tokens within the chunk, rather than the sentence level, to effectively reduce redundancy. Moreover, to overcome the long sequence issue, Li et al [58] comparably proposed Clinical-Longformer and Clinical-Big Bird pretrained on the Longformer [58] and Big Bird [119] models, respectively. Both proposed models used sparse attention mechanisms and linear level sequence lengths to mitigate memory consumption, thus increasing long-term dependency to train extensive clinical notes.

Recent development has also introduced MEDVOC [64], which uses GPT architecture to improve the adaptation of vocabulary in medical texts. By efficiently reducing fine-tuning time, MEDVOC achieves competitive performance, with ROUGE scores of 51.49, 47.54, and 19.51 across different datasets, such as PubMed, BioASQ, and EBM, respectively.

#### Text Classification

The medical text classification task categorizes medical text datasets into predefined categories based on the content and context within the text [120]. Disease classification, medical image classification, drug classification, and sentiment analysis are some of the standard text classification applications in health care.

The key models are jpCR+jpW, BioMed-RoBERTa, ClinicalBERT, Mask-BERT, KG-MTT-BERT, EduDistilBERT, PathologyBERT, KD distilledBERT, MatSciBERT, and Bioformer.

Wada et al [120] proposed a BERT model, jpCR+jpW, that uses a classification method. The method pretrains the medical BERT model once following the up-sampling step of domain-specific word amplification. This is done to achieve better performance on a smaller medical corpus. Similarly, BioMed-RoBERTa [70] used the RoBERTA model and applied a domain and task-adaptive pretraining strategy with a simple data selection approach for domain-specific classification. By pretraining on domain-specific and unlabeled data, the model achieved 87% accuracy. ClinicalBERT [67] represents clinical notes effectively, with a word similarity accuracy of 90% to generate visualized and interpretable embeddings for capturing semantic associations between clinical texts.

Yogarajan et al [121] suggested applying multi-labels (eg, using more than 300 labels for longer documents) to enhance the performance of medical classification tasks. Furthermore, to solve the imbalance class problems, Rodrawangpai et al [122] demonstrated a framework of adding normalization layers and dropout to BERT-based models, which improved the classification performance by 4% on data that included imbalance target labels. Similar efforts have been made by Nguyen [50] to address label-abandoning problems in medical abstract classification. The author proposed that a BERT model with label attention in the fine-tuning process raised the F1 score by 0.3 and supported the explainability of the prediction results. Learning is difficult with insufficient labeled data in a low-resource experiment setting. To alleviate this problem, Mask-BERT [71] proposed a framework for few-shot learning, where the mask is applied to the input text and enables the gathering of more definitive tokens. Masked learning leads to filtered results on anchored samples from the data being used for representation, increasing the robustness of the output features.

Several language-specific models have been developed, including RuBioBERT and RuBioRoBERTa [123] for Russian text, BERTurk [124] for Turkish text, BioGottBERT for German text [88], and a Spanish text model [125], demonstrating the applicability of BERT-based models beyond English. Moreover, various disease-specific classification models have been developed. For example, PathologyBERT [126] is a pretrained masked language model used for classifying the severity of breast cancer diagnoses, raising the importance of applying domain-specific tokenization. KD distilled_BERT [127] is a response-embedded knowledge distillation framework that used pretrained BERT for depression classification and achieved a high accuracy of 97%. MatSciBERT [68] presents a biomedical domain-specific classification model on abstracts of the literature with binary classification application. The model extracted the context of the embeddings alongside the topic and had 2.75% higher accuracy than SciBERT.

To overcome the over-fitting and dimensionality problems for extracting numerous features in the text classification task, AFKF [128] proposed a fusion block with Kalman filters onto features of EMRs. This led to a 20% increase in accuracy compared with previous models. Likewise, to classify the features in EMRs, BERT-MSA [129] showed that a multilayered self-attention mechanism improved accuracy in obtaining relevant features. EduDistilBERT [130] demonstrated that adapting a smaller BERT model with limited parameter usage increases overall performance by 95% while reducing the computation cost. Another BERT-based fusion approach by Al‑Garadi et al [131] explored architecture to fuse BERT, ALBERT, and RoBERTa model probabilities using a naive Bayes classifier, achieving an F1 score of 0.67 in classifying medication abuse texts.

Recently, the Bioformer [69] model demonstrated a 60% reduced model size and a 2- to 3-fold increase in performance speed. The model used a whole-word masking approach with 15% masking, which provided contextual information. However, KG-MTT-BERT [72] raised a question on limitations for multi-type clinical text classification. Concatenating numerous texts may be more efficient in developing relevant contextual information, and using only BERT may misplace crucial details. Therefore, the model extended the BERT model with a knowledge graph during fine-tuning, demonstrating effective handling in classifying patients into diagnosis-related groups.

Although the models were pretrained on the BERT model, Gao et al [132] showed that BERT-structured models did not gain better accuracy on clinical classification tasks, such as classifying discharge summaries or pathology reports, compared with nontransformer language models. Gao et al asserted that, in addition to the knowledge obtained through the entities, grammar patterns should also play a role in the model’s mechanism. Furthermore, beyond the applications mentioned above, text classification can be used with other tasks. For example, Wang et al [133] applied a question-answering task along with the classification task by using the BERT model to classify texts in question inputs from patient inquiries regarding their symptoms.

Recent advancements in text classification models include TransformEHR [73], which uses BERT and longitudinal EHR data for clinical disease prediction, achieving area under the receiver operating characteristic curve and area under the precision-recall curve scores of 81.95 and 78.64, respectively. MeDa-BERT [74] tailored embeddings for Danish medical text, with accuracy ranging from 86.7% to 97.1%. SCHOLARBERT [75] leveraged public resource-driven datasets for scientific NLP, obtaining an F1 score of 85.49%. MediGPT [76] improved medical text classification tasks, with accuracy and F1 scores of 90.0% and 88.7%, respectively, showing a 22.3% performance gain over traditional methods.

#### Sentiment Analysis

The sentiment analysis task captures and identifies expressions and opinions [134] in medical contexts, including clinical notes, social media posts related to medicine, or patient feedback. For instance, sentiment analysis can capture the perception of people expressed in social media during the COVID-19 outbreak [135-137]. Emotions, such as positive, neutral, and negative sentiments, expressed by the public dominated during the pandemic [138]. Additionally, multi-label sentiment classification proved that the BERT model provided better performance compared with the LSTM model [139]. Moreover, the opinions of patients and physicians can be used to describe the symptoms and diagnosis to facilitate the decision-making process and support the decisions in clinical patterns [77]. The primary goal of sentiment analysis in health care is to provide insights into patient experiences, such as attitudes toward health care services and overall medical experience satisfaction. It not only assists patients but also supports clinicians to identify any underlying issues in patient care.

The key models are MentalBERT, MeentalRoBERTa, SINA-BERT, SGTN, RedBERT, T-BERT, AKI-BERT, TopicBERT, TweetBERT, and BelabBERT.

In mental health, patients’ written texts have become a valuable source for supporting hypotheses and providing insights into the emotions expressed by patients [85,140]. While more research using PLMs needs to be conducted in this field, MentalBERT, MentalRoBERTa [77], PsychBERT [140], and belabBERT [85] applied mental health texts and achieved sentiment classification accuracies of 75%, 86%, and 90%, respectively. Additionally, transformer language models have been studied for sentiment analysis in languages other than English. Some language models are being developed to accommodate the unique structure and characteristics of different languages.

To achieve an effective model adaptation, researchers have explored HeBERT and HebEMO [141] for Hebrew, AraBERT and MARBERT [142] for Arabic, SINA-BERT [78] for Persian, and Fine-tuned BERT [143] for Chinese. However, few studies have conducted disease-specific sentiment analysis. RedBERT [80] involved a sentiment model for COVID-19, where BERT was used for classifying sentiments of Reddit comments to grasp insights into the pandemic. Mao et al [82] proposed AKI-BERT, where the model was developed to support the early prediction of acute kidney injury.

Social media data are often used as a source for medical sentiment analysis as they are more informal and conversational, making them useful for modeling the nuances of language models. The COVID-TWITTER-BERT model [144], which was pretrained on Twitter messages regarding COVID-19, showed improved performance on COVID-19–related datasets. In particular, TweetBERT [84] exhibited improved performance on COVID-19–related and biomedical datasets. TwitterBERT was evaluated on 12 different biomedical datasets and outperformed previous BERT models, such as SciBERT [113] and BERT [145].

Comparably, TopicBERT [83], a memory-efficient BERT model, fine-tuned and enhanced sentimental analysis performance, and a complementary topic framework was applied to improve its performance. Beyond the proposed frameworks presented above, AlBadani et al [79] proposed a graph transformer model, SGTN, which used BERT to pretrain node embeddings and aggregated neighboring information to efficiently learn sentiments. It showed 5% improvement over baseline models.

#### Named Entity Recognition

The NER task identifies the named entities in unstructured text data. In health care, NER is used to automatically extract and define relevant medical entities, including diseases, medications, procedures, and other clinical concepts, from medical texts in research papers or EMRs [146,147]. The common applications of NER in medicine are as follows: (1) identify and analyze medical entities and relationships in medical literature to support biomedical findings [148]; (2) extract patient data, such as diagnosis, medication, laboratory results, and physical measurements, from EMRs to improve the decision-making for clinicians and the overall care [149]; and (3) extract and categorize data from medical claims and hospital admission and discharge data to improve health care management and resource allocation [150,151].

The key models are Bio+ClinicalBERT, Med-BERT, G-BERT, BioALBERT, GatorTron, ELECTRAMed, CamemBERT, BioGottBERT, Ra-RC, and RG-FLAT-CRF.

Numerous language models have been developed to implement EMR or EHR data for NER tasks in the clinical field. Bio+Clinical BERT [61] achieved superior results in clinical texts, with an F1 score of 83%. While the ClinicalBERT and Clinical BioBERT models were trained on EHRs, the Bio+Clinical BERT model did not perform well on deidentification text. Other models, such as G-BERT, also used EHR data to propose a language model that combined graph neural networks and BERT for representing medication information and predicting drug recommendations [87]. MedGPT [96] effectively processed noises by organizing medical text in multi-step procedures. In the first stage of the proposed model, unstructured data were converted into a standardized ontology using NER+L. Then, GPT was used for forecasting diagnosis events.

Moreover, studies attempted to tackle problems regarding representing and learning long medical entities [94,152]. Liu et al [152] proposed Med-BERT, using a Span-FLAT method for longer medical entities, and it achieved an F1 score of 84%. By contrast, the BIBC model built by Yang et al [94] captured both local and global sequence features to efficiently solve long text input issues. Additionally, models were trained on an extensive collection of biomedical texts to overcome the limited amount of training data. For example, BioALBERT and GatorTron attempted to develop a large medical language model [65,93]. BioALBERT used vocabulary specifically tailored to the biomedical domain and applied the ALBERT structure. On the other hand, GatorTron used the byte pair encoding algorithm and was pretrained on the GPT model to scale up the language model up to 8.9 billion parameters, showing 9.6% accuracy improvement. Furthermore, the datasets in the medical domain face the challenge of not only limited training data but also low-quality labeled training data. Therefore, multi-task learning was presented by Khan et al [92], and the slot tagging problem was approached with MT-BioNER, a multi-task transformer-based model that enhanced memory performance and time efficiency in slot tagging, with 10% better performance than single-task models.

While recent studies have heavily relied on BERT-based structures, transformer models used other PLMs for improving NER tasks. ELECTRAMed [91] proposed an ELECTRA-based model for the biomedical domain, which reduced the sequence length and training phases. Additionally, many models have focused on multilingualism, including the CamemBERT, BioGottBERT, Ra-RC, and RG-FLAT-CRF models [95,153,154], focusing on efficiently learning features in languages other than English, such as French, German, and Chinese.

More recent studies include HeartBERT [98], which emphasizes department-specific models, focusing on cardiology and achieving 74% accuracy. UMLS-KGI-BERT [99] introduced graph-based learning for clinical text extraction, with a precision of 85.05%. CardioBERTpt [100], which is specialized in Portuguese cardiology terms, improved NER performance, with an FL-score of 83%. Finally, TocBERT [101], which is fine-tuned on the MIMIC-III dataset, outperformed rule-based methods for segmenting discharge summaries, achieving an F1 score of 84.6%.

## Discussion

### Principal Findings

This study examined previous studies on transformer-based language models in the medical domain. We reviewed a total of 75 recently studied models that aligned with our inclusion criteria. The initial step of the method involves categorizing the models based on the tasks they perform, such as dialogue generation, question answering, summarization, text classification, sentiment analysis, and NER. Then, each study is analyzed based on the key findings, frameworks, pretraining models used, and model names. Finally, the limitations of each task application are discussed. The use of transformer-derived language models in medicine has shown numerous advantages, such as high accuracy, language comprehension, automated diagnosis, adaptability, and efficiency. However, these models also face several challenges, including the lack of standardization, the need for domain-specific knowledge, limited annotated training data, safety concerns, interoperability and interpretability issues, integration complexities, ethical considerations, and evaluation issues. In this discussion section, we explore key limitations and future directions of models in terms of each task and the generalizability of the explored models across different health care settings and population considerations. Model challenges, their potential solutions, and the future of natural language models in the medical domain will be discussed.

### Specific Task-Based Challenges and Future Directions

#### Dialogue Generation

Dialogue generation models like DialoGPT and ChatDoctor face challenges such as handling the complexity and specificity of medical terminology, ensuring data privacy, and providing accurate, contextually relevant, and empathetic responses. Privacy and security issues are critical since these models deal with sensitive patient information. The risk of privacy, chances of errors, ethical constraints, and security issues remain to be addressed. The challenge comes from the specificity and complexity of medical terminology, although the medical dialogue system certainly should provide only accurate and informative knowledge tailored to the level of expertise of the end user. Therefore, human experts need to conduct regular risk and security audits.

The following suggestions are made to further improve medical dialogue research. First, continuous learning and training of the dialogue system are necessary to incorporate up-to-date knowledge for users. Additionally, language translation could be integrated into the dialogues to enable universal access to data and promote a more profound exchange of insights without language barriers. Moreover, chatbots [107] should be integrated in a real medical setting to reduce medical costs and physician burdens. Proper and accurate usage of the dialogue system may assist patients in navigating through the vast amount of freely available online data, finding correct information, and avoiding falsified or unsolved answers. Lastly, automated data augmentation techniques can be used to create unbiased dialogues. These suggestions can lead to further advancements in medical dialogue research, leading to more efficient communication between patients and medical professionals.

#### Question Answering

Medical question-answering systems like BioBERT and UmlsBERT struggle with the complexity of medical terminology for nonexperts in the medical field. Patients who are experiencing an illness may find it difficult to filter and search for relevant information. These models need to handle diverse linguistic data and adapt regional variations in medical practices. One approach to addressing these limitations is to integrate multilingual models to handle questions in various languages. Another approach is the incorporation of region-specific medical data to improve model generalizability and accuracy. Further, enhancing the ability to integrate summarization tasks on top of the question-answering system may provide comprehensive responses. However, such a multi-task system requires several human experts to evaluate the provided answers in order to judge the task performance accurately.

#### Summarization

Medical summarization is a crucial application in language model tasks to facilitate the hospital’s process and significantly reduce the workload and burnout of clinicians. However, challenges emerge due to the complexity of health care terminologies and the need for expert knowledge to comprehend them. The ability to achieve concise and faithful summaries is critical for avoiding physician burnout and patient dissatisfaction. Models like BioBERTSum and ClinicalBERTSum face challenges in learning sentence and document-level features, handling complex medical terminologies, and ensuring summaries are concise and accurate. The risk of physician burnout due to extensive documentation can be mitigated by effective summarization. Future work can focus on developing a system of human expert assessments to validate the summarization quality. Additionally, combining extractive methods and abstractive summarization methods is suggested. A fine-tuned summarization model for a particular task should consider tense information and personal information. We recommend building an ensemble method to improve pretraining and fine-tuning datasets for summarization effectiveness. Medical summarization is a crucial application in language model tasks to facilitate the hospital’s process and significantly reduce the workload and burnout of clinicians.

#### Text Classification

Improving the accuracy and effectiveness of classification tasks poses several challenges and limitations that need to be addressed. Models, such as BioMed-RoBERTa and ClinicalBERT, need to address issues related to class imbalance, limited annotated data, and the complexity of medical terminologies. Limited training data, for instance, can be addressed by collaborating with different institutions to gather various information options in vocabulary usage and text structure, and high-quality annotated data can thus be developed. Ambiguity, variation, concept drift, data privacy, language complexity, and class imbalance can be addressed by employing domain-specific approaches, and pretraining language models can be leveraged on similar datasets. Domain-specific approaches can resolve ambiguity issues and achieve active learning to reduce the reliance on large volumes of labeled data. These strategies will facilitate better model performance results.

#### Sentiment Analysis

Despite previous research results, medical sentiment analysis remains a challenging task due to personalized information required to accurately measure meaning and interpret emotions in context. MentalBERT and RedBERT, for instance, need to accurately interpret emotions in medical contexts, handle personalized information, and manage the complexity of evaluating representations in the biomedical domain [155]. Organizing emotions in context requires sentiments, including sarcasm, emojis, and misspelled words, which create subjectivity, as noted by Brezulianu et al [156].

These limitations can be overcome by defining emotional polarity for annotations and integrating cultural, economic, and medical contexts into the model. Future research should consider using a domain-specific sentiment dataset, adapting the specific medical source (despite the lack of an available dataset, mostly from a single source), creating highly effective and defined labels in data, performing analysis based on the context, building both the explicit and implicit sentiment lexicon, and addressing the lack of a mental health–related sentiment lexicon. By addressing these challenges, future work can develop more accurate and effective medical sentiment analysis language models.

#### Named Entity Recognition

NER downstream tasks are imperative to address its limitations. The limited annotated data in medical text datasets is a major challenge due to the high cost and time involved in labeling, resulting in limited labeled data for model training. The Bio+ClinicalBERT and Med-BERT models face challenges in handling limited annotated data, normalizing various terminologies, and ensuring accurate entity extraction across different medical texts.

Therefore, we suggest collaborative effort among health care providers, biomedical researchers, and computer engineering experts to develop effective and robust NER models. Improving the annotation algorithms and creating extensive and accurately labeled medical text datasets can significantly enhance the performance. Moreover, standardized clinical entities can prevent ambiguity arising from abbreviations and context. The use of transfer learning techniques and domain PLMs can be beneficial in addressing the limited annotated data issue. Developing domain-specific dictionaries and ontologies can aid in improving the model performance.

### Generalizability Challenges

Health care systems vary widely in their practices, protocols, and terminologies. For instance, a model trained on data from the United States may not perform optimally in a health care setting in Asia or Europe due to differences in clinical infrastructure and settings. The availability of resources, such as EHRs and technological infrastructure, can also differ between urban and rural settings, and between developed and underdeveloped countries. This variability can significantly affect the implementation and performance of the models.

Moreover, patients from different ethnic and cultural backgrounds may present symptoms differently and may have varying health behaviors, and models need to account for these varying characteristics to avoid biases and ensure equitable health care delivery. Moreover, multilingual populations pose a challenge for language models trained predominantly on English language data. The inclusion of diverse linguistic data during model training can mitigate this issue with a language-specific pretraining stage followed by a shared fine-tuning stage to improve the model’s applicability across different regions.

By incorporating diverse datasets during training, language models can support personalized medicine initiatives. This involves tailoring medical treatments to individual patient characteristics, leading to more effective and efficient care. Developing adaptable models that can be fine-tuned with local data ensures scalability across different health care settings, to address regional variations in medical practices and patient demographics.

### Standardizing Medical Data for Improved Model Performance

The quality and consistency of medical data may vary across health care settings. Models trained on high-quality standardized data may not perform as well when applied to settings with less structured and lower quality data. The lack of standardized terminologies in medical texts, which encompass a vast array of terminologies from disease-specific to domain-specific language, is a notable challenge. Currently available datasets often have a restricted range of medical entities, posing difficulties in accurately extracting relevant entities.

To address this, we suggest creating standardized clinical entities. This would enable the normalization of different names or abbreviations to accurately normalize entities for standard medical terminology, thereby improving data consistency and model performance. Building standardized forms that are widely adopted and available in multiple languages will facilitate standardized medical learning. Additionally, developing domain-specific models has proven effective in enhancing model performance. For instance, an open-source package for detecting clinical entities from medical texts, which can recognize risk factors, medications, and diagnoses, can be developed to support this initiative.

The fuel of building and training language models is data. Collecting accurate information and precisely fabricating the data design during the preprocessing step is crucial. The challenges in creating such quality data for medical language models include a lack of key annotation and limited training data. Annotating medical text is time-consuming and costly, resulting in limited labeled data for training models. First, using diagnosis codes on weak supervision for training labels is suggested. Second, the pipeline should support the automated retrieval of datasets and multiple types of clinical entities to enable the preservation of annotation relationships across different languages. The automated retrieval of datasets and the development of speedy and supportive algorithms can aid in data integration and preprocessing. In addition to technical solutions, emphasizing the importance of multidisciplinary collaboration can significantly enhance the development and implementation of these models. By integrating expertise from various fields, we can overcome challenges, develop innovative solutions, and further advance the field of AI in health care. Collaborative efforts among data scientists, clinicians, bioinformaticians, and ethicists are crucial for building robust, reliable, and ethically sound models.

### Ethical Considerations

Interoperability and cybersecurity pose significant challenges in medicine. EMRs and clinical decision support systems often have difficulty interacting with each other, leading to inefficiencies in patient care. To overcome these challenges, it is important to develop strategies focused on informed consent, safety, transparency, and algorithmic fairness for bias prevention. Ensuring that patients provide informed consent for the use of their data is critical. This involves informing patients about the use of their data, the benefits and risks, and their rights to withdraw consent at any time. This process upholds patient autonomy and enhances trust in AI systems.

Safety and transparency are fundamental to the ethical deployment of AI models in health care. Models handling sensitive patient data and providing clinical recommendations, such as BioBERTSum, must be rigorously validated and continuously monitored to detect and rectify errors promptly. Transparency can be achieved by making algorithms and decision-making processes understandable to users. This includes documenting how models are trained, the types of data used, and the underlying mechanisms of the algorithms.

Ensuring algorithmic fairness is crucial to prevent biases in AI models, which could lead to unequal treatment of patients. AI models trained on biased datasets can perpetuate existing disparities in health care. For example, models must include diverse and representative data to avoid underrepresentation of certain populations, ensuring fairness and accuracy across different groups such as ethnicity, cultural background, gender, and age. By addressing these ethical considerations within mitigated guidelines, we can ensure the reliability of transformer language models in medicine to improve overall health care while preserving fairness.

### Evaluation Metrics

Furthermore, the rapid evolution of medical knowledge poses a challenge for language models to adapt and remain up to date with innovative discoveries. Ensuring the interpretability of language models is also crucial to address the trust issue and support the decision-making process. To evaluate medical language models, multiple metrics, including the F1 score, Biomedical Language Understanding Evaluation, Biomedical Language Understanding & Reasoning Benchmark (BLURB), and Chinese Biomedical Language Understanding Evaluation [157], should be used to overcome unbalanced performance issues.

### Conclusion

We presented a comprehensive survey of task-specific transformer-derived models employed for diverse medical tasks, demonstrating their significant potential in the medical domain. Numerous studies have highlighted their capabilities in improving health outcomes, extending beyond disease prediction and medical classification studies. Our work clearly delineates the applications of transformer-based language models in various medical tasks such as dialogue generation, question answering, summarization, text classification, sentiment analysis, and NER. We identified innovative models and their unique contributions to the field. These findings distinguish our work from existing literature by providing a detailed, task-specific analysis of transformer-based models in health care.

Despite the promising advancements, several challenges must be addressed to develop effective models. These include standardization, limited annotated data, interoperability, and ethical considerations. To overcome these challenges, it is crucial to emphasize multidisciplinary collaboration. Future research should investigate transformer models that incorporate visual or audio data sources to provide a more comprehensive understanding of medical contexts.

Developing models that support patients’ experiences and assist health care practitioners in focusing solely on critical tasks by providing evidence-based recommendations and identifying potential diagnostic and treatment options can remarkably improve patient care. AI-driven tools rationalize administrative tasks, reduce paperwork, and improve workflow efficiency, eventually saving time for health care providers. Further, policymakers can leverage insights from transformer-based models to inform health care policies and allocate resources more effectively, ensuring equitable health care delivery.

This review solely focused on transformer language models that used text data. While the findings are promising, the applicability of these models may vary across different medical settings and populations. Our findings highlight the transformative potential of transformer-based language models in the medical field. By addressing the identified challenges and focusing on innovative research directions, the health care domain can advance significantly. We encourage researchers to build upon our work, address these challenges, and explore new frontiers in medical AI to improve patient care and clinical decision-making.

## Appendix

Multimedia Appendix 1 PRISMA (Preferred Reporting Items for Systematic Reviews and Meta-Analyses) checklist.

## Abbreviations

AI: artificial intelligence
BERT: Bidirectional Encoder Representations from Transformers
EHR: electronic health record
EMR: electronic medical record
GPT: Generative Pre-trained Transformer
NER: named entity recognition
NLP: natural language processing
PLM: pretrained language model
